# Illegal, Unreported, and Unregulated Fisheries Threatening Shark Conservation in African Waters Revealed from High Levels of Shark Mislabelling in Ghana

**DOI:** 10.3390/genes12071002

**Published:** 2021-06-29

**Authors:** Narkie Akua Agyeman, Carmen Blanco-Fernandez, Sophie Leonie Steinhaussen, Eva Garcia-Vazquez, Gonzalo Machado-Schiaffino

**Affiliations:** Department of Functional Biology, University of Oviedo, 33006 Oviedo, Spain; narkieagyeman@gmail.com (N.A.A.); carmen.mbfy@gmail.com (C.B.-F.); sophie.steinhausen@imbrsea.eu (S.L.S.); egv@uniovi.es (E.G.-V.)

**Keywords:** DNA barcoding, mislabeling, seafood traceability, fisheries, elasmobranchs

## Abstract

Mislabelling of fish and fish products has attracted much attention over the last decades, following public awareness of the practice of substituting high-value with low-value fish in markets, restaurants, and processed seafood. In some cases, mislabelling includes illegal, unreported, and unregulated (IUU) fishing, contributing to overexploit substitute species that are undetectable when sold under wrong names. This is the first study of DNA barcoding to assess the level of mislabelling in fish marketed in Ghana, focusing on endangered shark species. Genetic identification was obtained from 650 base pair sequences within the cytochrome c oxidase I (COI) gene. All except one of 17 shark fillets analysed were wrongly labelled as compared with none of 28 samples of small commercial pelagic fish and 14 commercial shark samples purchased in Europe. Several substitute shark species in Ghana are endangered (*Carcharhinus signatus* and *Isurus oxyrinchus*) and critically endangered (*Squatina aculeata*). Shark products commercialized in Europe (*n* = 14) did not reveal mislabelling, thus specific shark mislabelling cannot be generalized. Although based on a limited number of samples and fish markets, the results that reveal trade of endangered sharks in Ghana markets encourage Ghanaian authorities to improve controls to enforce conservation measures.

## 1. Introduction

Fish and fishery products are amongst the most popular food commodities traded worldwide, accounting for up to 20.5 kg of per capita food fish consumption in 2018 [1]. More than half of the world’s population depends on fish as a source of protein, and the high supply coupled with inadequate resource management has led to depletion of global saltwater fish stocks [2]. Current fish stocks at unsustainable levels have increased from 10% in 1974 to 34.2% in 2017 [1]. Regulations by international organizations [3,4] and regional fisheries management organisations (RFMOs) have been designed to promote the sustainable use of marine resources. Likewise, efforts to ensure traceability in the seafood chain have been implemented, for example, Eco-labels [5]. However, despite these efforts, fraudulent activities such as illegal, unreported and unregulated (IUU) fishing and mislabelling of products are carried out by the fishing industry to meet the high demand for seafood [6]. Global seafood trade requires adequate labelling, but the availability and implementation of labelling regulations vary across countries. The strong evidence of mislabelling in processed and prepackaged seafood [7,8,9,10,11] has led to the use of genetic tools in authenticating seafood to detect IUUs. There is a clear need for authenticating seafood products, both from a conservation perspective and to ensure customers get value for their money but also to prevent health issues such as allergies [9].

Today, one of the main conservation concerns regarding marine fish is sharks. The increase of catches in global shark fisheries, coupled with climate change, destruction of marine environments from overfishing, ocean mining, and pollution through ocean waste dumping have led to a drastic decline in shark populations over the past decade [12,13,14]. About 73 million sharks are captured annually for their fins and meat [15,16], and due to their inability to sustain populations to meet this high consumption demand, the International Union for Conservation of Nature has listed most shark species as critically endangered, endangered, vulnerable or near threatened [17]. However, unrecognizable products of endangered species are sold under wrong names, which contributes to their hidden exploitation that continues unnoticed [18]—until DNA unravels their use as a substitute species. For example, Hellberg et al. [19] detected the vulnerable thresher shark *Alopias vulpinus* sold as mako shark jerky in U.S. markets. Another study in Brazil revealed undeclared exploitation of highly endangered angelshark species of the genus *Squatina* through DNA analysis of muscle fragments and carcasses [20].

Bornatowski et al. [18] pointed out a higher risk of shark mislabelling as IUU in developing countries where shark meat is commonly sold without proper identification. The need for authentication becomes even more evident in certain African countries, such as Ghana, that have no specific seafood labelling regulations. The Ghana Standards Authority General Labelling Rules of 1992 (L.I. 1541, [21]) is the only general labelling law in the country, where all the products are termed ‘food and drugs’. Moreover, Fishery Regulations (e.g., Fisheries Act 625 of 2002) do not address the labelling of seafood products; only fish of high economic value such as tuna, which is managed by the International Commission for the Conservation of Atlantic Tuna (ICCAT), have specific labelling regulations.

Ghana’s fisheries sector generates an estimated USD one billion in revenue yearly [22]. The artisanal fishery is the largest in the sector, accounting for 70% of the total volume of fish landed in the country [23], with a majority of the landings being sardinella, croaker, anchovy, and mackerel [24]. Commercially appreciated fisheries such as mackerel, sardinella, and anchovy are managed to a certain degree, but, for other species of high ecological value, there is no management plan for fisheries in Ghana, for example, for sharks [25]. Elasmobranchs and billfishes mostly caught by fishers in the coastal waters of Ghana include bigeye thresher-fin shark (*Alopias superciliosus*), blue shark (*Prionace glauca*), bull shark (*Carcharhinus leucas*), common thresher-fin shark (*Alopias vilpinus*), scalloped hammerhead shark (*Sphyrna lewini*), short-fin mako shark (*Isurus oxyrinchus*), common tiger shark (*Galeocerdo cuvier*), sand tiger shark (*Carcharias taurus*), and great white shark (*Carcharodon carcharias*) [26]. In their risk assessment of sharks under fisheries, Queiroz et al. [27] also identified similar shark species caught in coastal Atlantic waters. Shark fisheries in Ghana are not regulated mainly because the species are caught as bycatch, and the meat is mostly used as bait for higher commercial species such as tuna, anchovies, and mackerels [28]. However, as fish stocks decline, fishers are shifting their target to shark meat and fins that have economic value [28]. Today, shark products can reach expensive prices in Ghanaian markets [29].

DNA barcoding has become a widely used method for identifying species [30], marketed seafood (e.g., [7,9,11,31]), and indeed commercial shark products [19,20]. In addition, it has proven useful in conservation of highly commercial and vulnerable shark populations because of its ability to provide information on population structure [16]. Here, we use DNA barcoding to authenticate seafood products sold in Ghanaian markets for the first time with a focus on sharks and samples of other species for comparison. The risk of mislabelling is identified, and the conservation status of substitute shark species is taken into account to evaluate the threats of IUU fishing to the sustainability of shark fisheries. Expectations were the following: (a) A higher level of mislabelling in Ghana would occur for sharks than for more regulated fish such as sardinellas, anchovies, and mackerels [28]. (b) Following Bornatowski et al. [18] we would expect a higher level of shark mislabelling in Ghana than in countries with stricter labelling laws.

## 2. Materials and Methods

### 2.1. Sample Collection

To test Hypothesis a, samples of shark fillets (*n* = 17) were collected from different sellers at the Jamestown landing site for artisanal fishers (5°32′06.0′′ N, 0°12′32.6′′ W), to be sure that fillets represented different individuals. This market receives landings from a wide range of fishers from neighbouring villages, making it the ideal location for obtaining a variety of shark species from Ghanaian coastal waters. As random representatives of more appreciated commercial fish we analysed headless eviscerated samples of anchovy, croaker, sardinella, and mackerel (total *n* = 28), sampled from the Tema Newtown fish market (5°38′35.6′′ N, 0°01′01.8′′ E). This market also receives landings from many fishers operating all along the Ghanaian coast.

To test Hypothesis b, we analysed shark fillets (*n* = 11) and muscle samples cut from whole individuals in the selling point (*n* = 3) acquired from local fish markets in Asturias (northern Spain), during the beginning of 2019, for comparison with sharks from the Ghanaian market.

Details of samples are shown in Table 1. The labels of all products were recorded for each sample. Samples were frozen after collection and sent on ice in coolers to the laboratory in Spain, where they were stored in ethanol at 4 °C.

### 2.2. PCR Amplification and Sequencing

Genomic DNA was extracted from muscle tissues using the Qiagen DNeasy^®^ Blood and Tissue Kit (Qiagen, Hilden, Germany), following the manufacturer’s instructions. After DNA extraction, a 655 base pairs (bp) length fragment within the cytochrome c oxidase subunit I gene (COI hereafter) was amplified by PCR from each sample. Each reaction contained 0.5 µM of forward and reverse COI-Fish primers [32], 0.25 mM dNTPs, 2.5 mM MgCl2, 1× Buffer GoTaq^®^ Promega, 0.15 µL of GoTaq^®^ Polymerase (5 µ/µL), 2 µL of DNA in a final volume of 20 µL. PCR products were run in a thermal cycler (Model 2720, Applied Biosystems, Foster City, CA, USA) with the following program: initial denaturation step at 95 °C for 5 min followed by 35 cycles of denaturation at 95 °C for 30 s, annealing at 57 °C for 30 s, elongation at 72 °C for 30 s, and a final extension at 72 °C for 15 min.

PCR results were visualised by electrophoresis in 2% agarose gel stained with 2.5 μL SimplySafe™ dye (EURX^®^, Gdańsk, Poland). After amplicons purification, automated fluorescence sequencing was performed and both strands (forward and reverse) of each DNA fragment were sequenced.

### 2.3. Species Identification from DNA

Forward and reverse sequences were manually edited and aligned using the ClustalW tool in BioEdit [33]. For species identification, the consensus sequences were compared to reference sequences using Basic Local Alignment Search Tool (BLAST) (https://blast.ncbi.nlm.nih.gov/Blast.cgi, accessed on 10 June 2020) [34], and species assigned from the best match.

A phylogenetic tree was constructed from COI sequences in MEGA_X_10.1.8 using the maximum-likelihood statistical method and 10,000 bootstrap replicates inferred from the Hasegawa–Kishino–Yano model for the elasmobranchs tree, to obtain a visual depiction of the clustering across species. The best substitution model fit for both trees was calculated in MEGA. *Chimaera opalescens* (GenBank Accession number GU244534.1) and *Rhizostoma pulmo* (GenBank Accession number KY131238.1) were used as outgroups for the elasmobranchs and fish phylogenetic trees, respectively. Additional sequences of elasmobranchs obtained from GenBank were added to test the validity of the marker (Supplementary Table S2).

### 2.4. Mislabelling Data Analysis

In the case of mismatch between the stated name on a label and the genetically identified species, the sample was considered mislabelled. A comparison of samples for mislabelling frequency was done using contingency Chi-square test with free PAST software version 3 [35].

In the group of mislabelled samples, the conservation status of substitute and label species was checked. We compared the distributions of conservation categories using the Chi-square test. Conservation status was obtained from the International Union for Conservation of Nature and Natural Resources (IUCN) Red List [36] and from the Convention on International Trade in Endangered Species of Wild Fauna and Flora (CITES).

## 3. Results

### 3.1. DNA Identification of Market Samples

Shark samples obtained from the Jamestown landing site were labelled as brown shark (*Carcharhinus plumbeus*) (*n* = 13), nurse shark (*Ginglymostoma cirratum*) (*n* = 1), bull shark (*Carcharhinus leucas*) (*n* = 1) and hammerhead shark (*Sphyrna* spp.) (*n* = 2). Shark samples from Spain were labelled as dogfish (“pintarroja” in Spanish, *Scyliorhinus* spp.) (*n* = 3) and blue shark (“tintorera” in Spanish, *Prionace glauca*) (*n* = 11). Commercial fish from the Tema fish market (Ghana) were labelled as anchovy *Engraulis* spp. (*n* = 10), mackerel *Scomber* spp. (*n* = 1), croaker *Pseudotolithus* spp. (*n* = 10), and sardinella *Sardinella* spp. (*n* = 7).

All the samples analysed were successfully identified using the COI marker (sequences between 221 and 694 bp; GenBank Accession numbers MW208696–MW208754). The list of species identified and GenBank accession numbers for their best match hit can be found in Supplementary Tables S1 and S2.

**Figure 1:**
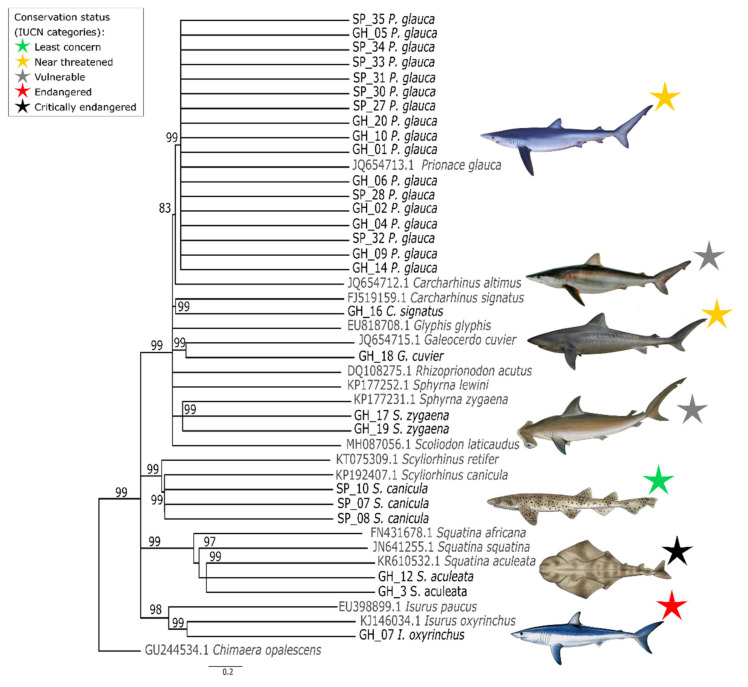
Phylogenetic tree based on COI sequences of shark species found in Ghanaian and Spanish fish markets, using *Chimaera opalescens* as an outgroup. The cut-off consensus value of >80% inferred from the maximum-likelihood method. Results of species clustering corresponding to 10,000 bootstrap replicates were obtained from the nearest-neighbour interchange using the Hasegawa–Kishino–Yano parameter model in MEGA X. GH, Ghana and SP, Spain. Individuals included in this study are highlighted in bold.

Seven species of sharks were identified from COI sequences using BLAST. *Prionace glauca* was found from Ghana and Spanish markets and accounted for the majority of samples (20 samples, 64.5%), followed by *Scyliorhinus canicula* (9.6%), *Isurus oxyrinchus*, *Sphyrna zygaena*, *Squatina aculeata* (6.5% each), and *Carcharhynus signatus* and *Galeocerdo cuvier* (3.2% each). Since some sequences were shorter than the expected fragment of 655 bp, a phylogenetic tree based on COI sequences was constructed to confirm BLAST results for shark species (Figure 1). For this purpose, sequences were trimmed down to 509 bp. All the sequences identified as the same species from BLAST clustered together, confirming BLAST results. The shape of the tree reconstructed using maximum-likelihood phylogenetic analysis was in concordance with well-defined shark phylogenies, such as those described by Cunha et al. [37] and Pavan-Kumar et al. [38]. Thus, shark barcoding with the molecular marker employed in this study can be considered to be reliable.

**Figure 2:**
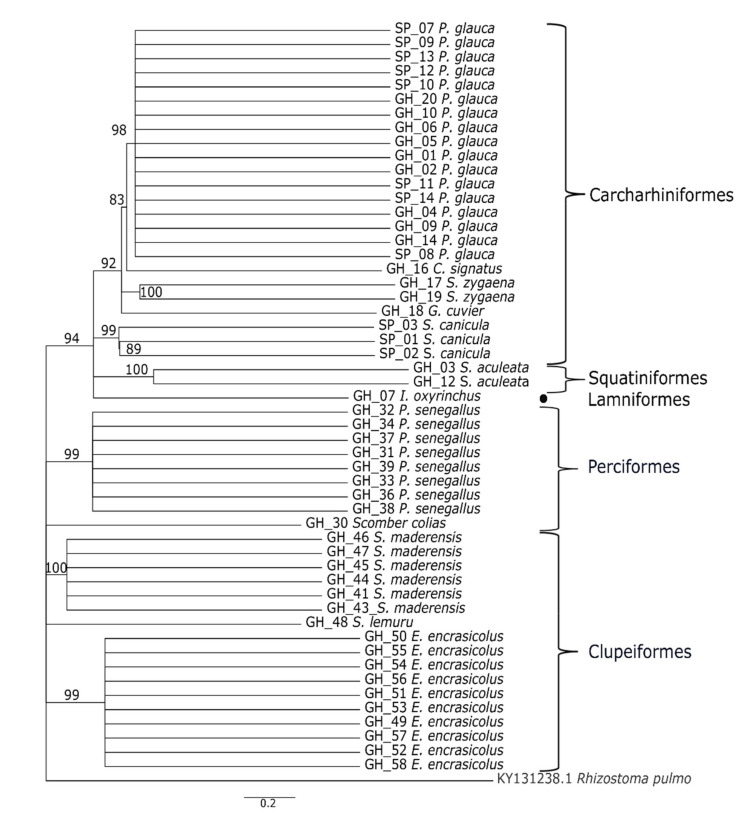
Phylogenetic analysis of COI sequences obtained from all fish samples (sharks, anchovy, mackerel, sardinella, croaker, and *Rhizostoma pulmo* as an outgroup) with the cut-off consensus value of >80% inferred from the maximum-likelihood method. Results of species clustering corresponding to 1000 bootstrap replicates were obtained from the nearest-neighbour interchange using the Kimura-2 parameter model in MEGA X. GH, Ghana and SP, Spain.

The sequences obtained from other Ghanaian commercial species were blasted against GenBank, allowing us to identify all the croakers as *Pseudotolithus senegallus*, the sardinellas as *Sardinella maderensis* (*n* = 6) and *Sardinella lemuru* (*n* = 1), the anchovies as *Engraulis encrasicolus,* and the mackerel as *Scomber colias*. The phylogenetic tree constructed from these COI sequences was clearly consistent with their taxonomic positions, with all correctly clustered by orders (Figure 2).

### 3.2. Mislabelling Analysis

A high level of mislabelling was found in commercial sharks from Ghana (Table 1): 94% of the shark fillets analysed were wrongly identified (16 out of 17 samples). Among the 13 samples that were labelled as brown shark, there were none that were *Carcharhinus plumbeus*, instead, they were four different species, i.e., *Carcharhinus signatus* (night shark), *Isurus oxyrinchus* (short-fin mako shark), *Prionace glauca* (blue shark), and *Squatina aculeata* (sawback angelshark). The fillet that was suppose to be nurse shark was genetically identified as *Sphyrna zygaena* (hammerhead shark), another labelled as bull shark was identified as *Galeocerdo cuvier* (common tiger shark), and one hammerhead shark was a blue shark *Prionace glauca*. There was ony one fillet analysed that was correctly labelled as hammerhead, since the species genetically identified was *Sphyrna zygaena*.

In contrast to the high level of mislabelling in sharks, the other marketed fish analysed, more appreciated in Ghana, were not mislabelled. Croakers, anchovies, sardinellas, and the mackerel sample DNA barcoded corresponded to species of the genera stated in the labels (Table 1): *Pseudotolithus senegallus* (law croaker), *Engraulis encrasicolus* (European anchovy), and *Scomber colias* (Atlantic chub mackerel). Sardinella samples were identified from DNA as two different species: one individual as *Sardinella lemuru* (Bali sardinella) and the other six as *S. maderensis* (Madeiran sardinella), which were not differentiated on the labels. Although there was no species substitution, labels were incomplete without the specific scientific name and could not be considered correctly labelled *sensu stricto*; however, although incomplete, following our definition above this was not mislabelling but incomplete or incorrect labelling. Mislabelling in sharks was indeed significantly higher than the absence of mislabelling found in the other commercial species analysed (χ^2^ = 40.89, 1 df, *p* < 0.001). Even taking into account that shark samples were less recognizable than the other species (only partially recognizable), this result seems to confirm Hypothesis a.

None of the sharks analysed from northern Spain were mislabelled (Table 1). The three pieces cut from whole individuals labelled as dogfish *Scyliorhinus* spp. were genetically identified as *Scyliorhinus canicula* (lesser spotted dogfish). As in the case of commercial fish from Ghana, these samples were incompletely labelled in the absence of the specific scientific name, but not mislabelled. The eleven frozen fillets of bluefish were authenticated as *Prionace glauca* from DNA. The difference between sharks sampled from Ghana and Spain was statistically significant (χ^2^ = 27.23, 1 df, *p* < 0.001). Even removing the three samples of dogfish that could be visually identified since the whole individual was available, the difference was still significant (χ^2^ = 24.16, 1 df, *p* < 0.001). This confirmed Hypothesis b.

### 3.3. Conservation Issues

According to CITES and the IUCN (Table 2), all the sharks identified from DNA in this study, except *Scyliorhinus canicula* (sampled from Spain), are decreasing globally. Considering the information displayed in the labels for the group of mislabelled sharks (*n* = 16), 6.25% were near threatened (one supposedly and the rest vulnerable). However, 62.5% of DNA-authenticated sharks were near threatened, while 6.25% were vulnerable, 18.75% endangered, and 12.5% critically endangered (*Squatina aculeata*). The distribution of individuals in conservation categories between labels and DNA-authenticated samples was significantly different (χ^2^ = 24.6, 3 df, *p* < 0.001). 

In addition, the croakers found in this study (*Pseudotolithus senegallus*) and the *Sardinella maderensis*, which are among the leading traded fish species in Ghana, are listed by the IUCN as vulnerable. *Sardinella lemuru* is also listed as near threatened, with decreasing global populations.

## 4. Discussion

To the best of our knowledge, this is the first study in Ghana to use molecular techniques to identify fish mislabelling in the country. Together with South Africa [7,39], Egypt [40], and Guinea-Bissau [41], this study joins the few studies performed in African countries that have applied DNA barcoding for the identification of species. Even from limited sample sizes and number of fish markets, the results showed a high level of mislabelling in sharks, while not so high in other fish species. This difference may be explained because sharks are not managed while pelagic fish are [25], thus, the latter are more controlled as discussed below. From a review by Bornatowski et al. [18], shark mislabelling is expected to be high in developing countries; our study confirmed this observation in Ghana.

Regulations for fishery resources in Ghana vary across species, with significant interest in commercial fish species [25]. Sardine, mackerel, croaker, anchovy, shrimp, and tuna are Ghana’s dominant commercially landed fish species, accounting for 70% of total marine production [29]. Due to their high economic importance in export value to the country, catch quotas are set by the Ministry of Fisheries and Aquaculture Development (MoFAD) for these resources, and fish population trends are observed [42]. This control might explain why no mislabelling was found in these species. In contrast, there is no total allowable catch (TACs) rates set for sharks that are mostly caught as bycatch and represent 1.6% of annual fish catch [25], The Fisheries Act 625 of 2002 and the Fisheries Regulation of 2010 (L.I. 1968) provide a legal framework for the operations of shark fisheries in Ghana. Regardless of all of these provisions, including international regulations such as the Port State Measures Agreement (PSMA), management of shark fisheries in the country is poor. Surveys conducted by the Fisheries Scientific Survey Division of MoFAD show that catch data for sharks are mostly lacking; whenever available, species of different taxonomic groups are lumped together [25]. The high level of mislabelling (94%) observed in the shark samples from Ghanaian markets might be at least partly attributed to the lack of regulations on species fishing and conservation. Furthermore, differences in presentation between sharks (sold as fillets) and the rest of Ghanaian fish (sold as whole pieces) may also contribute to this high difference, since less recognizable products are usually more frequently mislabelled [43]. In any case, the high level of mislabelling found in shark products in our study in Ghana cannot be dismissed, since fillets is the usual presentation of shark-derived products in fish markets.

In addition, economic reasons could also explain higher mislabelling in shark species than in other Ghanaian fish. The species not mislabelled in this study are of low market price, fetching between USD 2–4 per kilo as compared with shark products that cost around USD 20–30 per kilo [29]. Declining fish stocks of the leading commercial fisheries make the trade of sharks a lucrative business for local fishers, being an alternative source of income due to the high demand for shark products. Another potential explanation may be a lack of knowledge of the traded species, given the morphological differences between mislabelled sharks grouped under a sample label, for example, brown shark (Table 1). This is of special concern as endangered species that are caught are not reported.

Another issue detected in our study was the use of a generic name for more than one species, seen in hammerhead shark and pelagic species in Ghana and in dogfish shark in Spain. Since there are no laws regarding the labelling of seafood products in Ghana, this is not a case of fraud; however, selling different species under a common name may have adverse consequences for resource conservation. The sale of products under generic names impedes the effective management of each individual species, creating room for oversight of vulnerable populations [44].

On the one hand, the high level of mislabelling found in shark species in Ghana is not unusual in other regions. A similar example of a large-scale sale of sharks under umbrella terms was found in the UK, where mislabelled species were identified as *Prionace glauca* [45]. In Greek shark samples, Pazartzi et al. [46] observed a high mislabelling rate of 55.81%, with *Scyliorhinus canicula* as one of the most mislabelled species. Almerón-Souza et al. [47] also reported high covered exploitation of *Prionace glauca* (23.8%) and *Sphyrna lewini* (22.2%) in Southern Brazilian fish markets. On the other hand, although high rates of mislabelling have been identified in various seafood [9,48,49,50,51], there was no mislabelling in the 14 shark samples analysed from northern Spain, regardless of presentation (fillets or whole pieces). Spain has strict labelling regulations for seafood products origin [52]. However, three samples (SP_01–SP_03) were labelled as *Scyliorhinus* spp., which goes against the labelling regulations of Spain ((EU) 1379/2013) stating that seafood labels should contain both “commercial designation” and “complete scientific name”.

The shark species identified in this study are traded globally. *Prionace glauca* is the most traded pelagic shark worldwide with global landings at 103,528 mt [53,54,55]. Overexploitation is reflected in a decreasing trend of its global catch [56]. *P. glauca*, has been listed in Appendix II of the Convention on the Conservation of Migratory Species of Wild Animals (CMS), since May 2020 [57], but at the time of sample collection there were no trade regulations for the species by CITES. On the other hand, finding IUCN Red listed species such as *Squatina aculeata* critically endangered, and *Isurus oxyrinchus* endangered, in Ghanaian fish markets is of great concern. The two species have been listed in Appendix II of the [57], since 2014. Regulations such as the setting of TACs by the EU have ensured fishing pressure on sharks and other endangered species was reduced in European waters. TACs for deep-sea sharks were set at zero in 2012 [58], and coastal species are monitored to improve data reporting. Thus, Ghana could adopt similar measures to protect its shark populations.

## 5. Conclusions

This case study shows the importance of DNA barcoding to control IUU fishing in marine fish, highlighting the necessity of applying molecular tools for preventing biodiversity losses in commercially exploited species. Although based on a limited number of samples and fish markets, this novel study shows a high level of mislabelling in sharks from Ghanaian waters. Several mislabelled species were endangered, critically endangered, or vulnerable. The gaps in catch reporting observed in Ghanaian shark fisheries need to be addressed and shark management improved. Public education on the conservation status of Ghanaian fish could enable consumers to make more sustainable choices. The results of this pilot study could be used to help with the implementation of effective regulations of shark and other commercial fisheries by the Ghana Government.

## Figures and Tables

**Table 1:** Mislabelling detected on market fish samples. Market and location of sample collection, original label (scientific name), sample ID, type of sample, number of products sold under the same label (*N*), and percentage of mislabelling for each product.

Market	Labelled as	Type of Sample	Sample ID	*N*	Genetic Identification	Mislabelling
Jamestown, Ghana	Brown shark (*Carcharhinus plumbeus*)	Fresh, filleted	GH_01, GH_02, GH_04-GH06, GH09-GH_10, GH_14	13	*Prionace glauca* (*n* = 8)	Yes, 61.5%
			GH_07 & GH_08		*Isurus oxyrinchus* (*n* = 2)	Yes, 15.4%
			GH_03 & GH_12		*Squatina aculeata* (*n* = 2)	Yes, 15.4%
			GH_16		*Carcharhinus signatus* (*n* = 1)	Yes, 7.7%
Jamestown, Ghana	Nurse shark (*Ginglymostoma cirratum*)	Fresh, filleted	GH_17	1	*Sphyrna zygaena* (*n* = 1)	Yes, 100%
						
Jamestown, Ghana	Bull shark (*Carcharhinus leucas*)	Fresh, filleted	GH_18	1	*Galeocerdo cuvier* (*n* = 1)	Yes, 100%
						
Jamestown, Ghana	Hammerhead shark (*Sphyrna* spp.)	Fresh, filleted	GH_19 & GH_20	2	*Sphyrna zygaena* (*n* = 1)	No
					*Prionace glauca* (*n* = 1)	Yes, 50%
Tema Newtown, Ghana	Mackerel *(Scomber* spp.)	Fresh, headless	GH_30	1	*Scomber colias* (*n* = 1)	No
Tema Newtown, Ghana	Croaker *(Pseudotolithus* spp.)	Fresh, headless	GH _31- GH_40	10	*Pseudotolithus senegallus* (*n* = 10)	No
Tema Newtown, Ghana	Sardinella *(Sardinella* spp.)	Fresh, headless	GH _42- GH_48	7	*Sardinella maderensis* (*n* = 6) *Sardinella lemuru* (*N* = 1)	No
Tema Newtown, Ghana	Anchovy *(Engraulis* spp.)	Fresh, headless	GH_49- GH_58	10	*Engraulis encrasicolus* (*n* = 10)	No
Asturias, Spain	Dogfish shark *(Scyliorhinus* spp.)	Fresh, whole	SP_01- SP_03	3	*Scyliorhinus canicula* (*n* = 3)	No
Asturias, Spain	Blue shark *(Prionace glauca)*	Frozen, filleted	SP_04- SP_06, SP_07- SP_14	11	*Prionace glauca* (*n* = 11)	No

**Table 2:** Conservation status of the shark species of this study according to IUCN; number of individuals in labels (NL); number of individuals authenticated from DNA (NA), and their percentage of use as a substitute species. * Not identified at species level in the label. Concern levels: Least concern < near threatened < vulnerable < endangered < critically endangered.

Species	IUCN Conservation Status	N_L_	N_A_	% of Substitutes
*Carcharhinus leucas*	Near threatened	1	0	-
*Carcharhinus plumbeus*	Vulnerable	13	0	-
*Carcharhinus signatus*	Endangered	0	1	100%
*Galeocerdo cuvier*	Near threatened	0	1	100%
*Ginglymostoma cirratum*	Vulnerable	1	0	-
*Isurus oxyrinchus*	Endangered	0	2	100%
*Prionace glauca*	Near threatened	11	20	55%
*Scyliorhinus canicula*	Least concern	3*	3	0
*Sphyrna zygaena*	Vulnerable	2*	2	50%
*Squatina aculeata*	Critically endangered	0	2	100%

## Data Availability

The data presented in this study are available in Supplementary Materials.

## Supplementary Materials

The following are available online at https://www.mdpi.com/article/10.3390/genes12071002/s1, Table S1: List of all samples included in the study, Table S2: BLAST details of identified species.
