# Supplementary material for: Illegal, Unreported, and Unregulated Fisheries Threatening Shark Conservation in African Waters Revealed from High Levels of Shark Mislabelling in Ghana
Source: Genes (Basel). 2021 Jun 29;12(7):1002. doi: 10.3390/genes12071002 (PMC8305889; doi:10.3390/genes12071002)
Supplement: Supplementary file 1 [file genes-12-01002-s001.zip › genes-1268622-supplementary.pdf]

Supplementary material

Supplementary Table S1. List of all samples included in the study. The original label on the package, species identified, GenBank accession numbers, final length in base pairs, the result of mislabelling, and conservation status of all samples are shown. GH= Ghana and SP=Spain.

Sample ID	Labelled as	Genetic identification	GenBank Accession Number	Length (bp)	Mislabelling	IUCN Status	CITES Listing
Ghana samples							
GH_01	Brown shark	*Prionace glauca*	MH194481.1	657	YES	Near threatened	Not listed
GH_02	Brown shark	*Prionace glauca*	KF590237.1	630	YES	Near threatened	Not listed
GH_03	Brown shark	*Squatina aculeata*	KR610532.1	626	YES	Critically endangered	Not listed
GH_04	Brown shark	*Prionace glauca*	KJ146042.1	599	YES	Near threatened	Not listed
GH_05	Brown shark	*Prionace glauca*	KJ146042.1	630	YES	Near threatened	Not listed
GH_06	Brown shark	*Prionace glauca*	KJ146042.1	630	YES	Near threatened	Not listed
GH_07	Brown shark	*Isurus oxyrinchus*	KJ146030.1	600	YES	Endangered	Appendix II
GH_08	Brown shark	*Isurus oxyrinchus*	KJ146030.1	570	YES	Endangered	Appendix II
GH_09	Brown shark	*Prionace glauca*	KJ146042.1	611	YES	Near threatened	Not listed
GH_10	Brown shark	*Prionace glauca*	KJ146042.1	631	YES	Near threatened	Not listed
GH_12	Brown shark	*Squatina aculeata*	KR610532.1	641	YES	Critically endangered	Not listed
GH_14	Brown shark	*Prionace glauca*	MH719984.1	577	YES	Near threatened	Not listed
GH_16	Brown shark	*Carcharhinus signatus*	FJ519159.1	599	YES	Vulnerable	Not listed

GH_17	Nurse shark	*Sphyrna zygaena*	MH194504.1	621	YES	Vulnerable	Appendix II
GH_18	Bull shark	*Galeocerdo cuvier*	MH911012.1	549	YES	Near threatened	Not listed
GH_19	Hammerhead shark	*Sphyrna zygaena*	MH194422.1	680	NO	Vulnerable	Appendix II
GH_20	Hammerhead shark	*Prionace glauca*	MH194480.1	641	YES	Near threatened	Not listed
GH_30	Chub mackerel	*Scomber colias*	KT074092.1	630	NO	Least concern	
GH_31	Croaker	*Pseudotolithus senegallus*	KP722769.1	630	NO	Vulnerable	
GH_32	Croaker	*Pseudotolithus senegallus*	KP722769.1	650	NO	Vulnerable	
GH_33	Croaker	*Pseudotolithus senegallus*	KP722769.1	630	NO	Vulnerable	
GH_34	Croaker	*Pseudotolithus senegallus*	KP722769.1	610	NO	Vulnerable	
GH_35	Croaker	*Pseudotolithus senegallus*	KP722769.1	620	NO	Vulnerable	
GH_36	Croaker	*Pseudotolithus senegallus*	KP722769.1	610	NO	Vulnerable	
GH_37	Croaker	*Pseudotolithus senegallus*	KP722769.1	630	NO	Vulnerable	
GH_38	Croaker	*Pseudotolithus senegallus*	KP722769.1	630	NO	Vulnerable	
GH_39	Croaker	*Pseudotolithus senegallus*	KP722769.1	640	NO	Vulnerable	
GH_40	Croaker	*Pseudotolithus senegallus*	KP722769.1	630	NO	Vulnerable	
GH_41	Sardinella	*Sardinella maderensis*	AP009143.1	630	NO	Vulnerable	
GH_43	Sardinella	*Sardinella maderensis*	AP009143.1	630	NO	Vulnerable	
GH_44	Sardinella	*Sardinella maderensis*	AP009143.1	630	NO	Vulnerable	
GH_45	Sardinella	*Sardinella maderensis*	AP009143.1	630	NO	Vulnerable	
GH_46	Sardinella	*Sardinella maderensis*	AP009143.1	620	NO	Vulnerable	

GH_47	Sardinella	*Sardinella maderensis*	AP009143.1	630	NO	Vulnerable	
GH_48	Sardinella	*Sardinella lemuru*	MT294005.1	630	NO	N/A	
GH_49	Anchovy	*Engraulis encrasicolus*	KP136718.1	630	NO	Least concern	
GH_50	Anchovy	*Engraulis encrasicolus*	KU056680.1	678	NO	Least concern	
GH_51	Anchovy	*Engraulis encrasicolus*	KP940607.1	670	NO	Least concern	
GH_52	Anchovy	*Engraulis encrasicolus*	KY176470.1	650	NO	Least concern	
GH_53	Anchovy	*Engraulis encrasicolus*	KP940607.1	674	NO	Least concern	
GH_54	Anchovy	*Engraulis encrasicolus*	MN893191.1	677	NO	Least concern	
GH_55	Anchovy	*Engraulis encrasicolus*	KU056680.1	676	NO	Least concern	
GH_56	Anchovy	*Engraulis encrasicolus*	KP940607.1	686	NO	Least concern	
GH_57	Anchovy	*Engraulis encrasicolus*	KP940607.1	679	NO	Least concern	
GH_58	Anchovy	*Engraulis encrasicolus*	KP940607.1	694	NO	Least concern	
Asturias Shark samples							
SP_01	*Scyliorhinus sp.*	*Scyliorhinus canicula*	KY949095.1	314	NO	Least concern	Not listed
SP_02	*Scyliorhinus sp.*	*Scyliorhinus canicula*	KY949053.1	385	NO	Least concern	Not listed
SP_04	*Prionace glauca* "Caella"	*Prionace glauca*	MN641801.1	278	NO	Near threatened	Not listed
SP_05	*Prionace glauca* "Caella"	*Prionace glauca*	MN641800.1	221	NO	Near threatened	Not listed
SP_06	*Prionace glauca* "Caella"	*Prionace glauca*	MN641800.1	236	NO	Near threatened	Not listed
SP_07	*Prionace glauca* “Tintorera"	*Prionace glauca*	MH719984.1	601	NO	Near threatened	Not listed

SP_08	*Prionace glauca* "Tintorera"	*Prionace glauca*	MH719984.1	591	NO	Near threatened	Not listed
SP _09	*Prionace glauca* "Tintorera"	*Prionace glauca*	MH719984.1	601	NO	Near threatened	Not listed
SP_10	*Prionace glauca* "Tintorera"	*Prionace glauca*	MH719984.1	601	NO	Near threatened	Not listed
SP_11	*Prionace glauca* "Tintorera"	*Prionace glauca*	MG703523.1	601	NO	Near threatened	Not listed
SP_12	*Prionace glauca* "Tintorera"	*Prionace glauca*	MH719984.1	591	NO	Near threatened	Not listed
SP_13	*Prionace glauca* "Tintorera"	*Prionace glauca*	MH719984.1	591	NO	Near threatened	Not listed
SP_14	*Prionace glauca* "Tintorera"	*Prionace glauca*	MH719984.1	601	NO	Near threatened	Not listed

Supplementary Table S2. BLAST details of identified species. E-value, identity percentage, total score and query cover of sequences submitted to GenBank. GH= Ghana and SP=Spain.

Sample	Genetic identification	Common name of identified species	E-value	Identity (%)	Score	Query cover %
Ghanaian Shark samples						
GH_01	*Prionace glauca*	Blue shark	0.0	99.85	1206	99
GH_02	*Prionace glauca*	Blue shark	0.0	100.00	1144	98
GH_03	*Squatina aculeata*	Sawback angelshark	0.0	100.00	1120	96
GH_04	*Prionace glauca*	Blue shark	0.0	99.00	1074	100
GH_05	*Prionace glauca*	Blue shark	0.0	99.52	1146	99
GH_06	*Prionace glauca*	Blue shark	0.0	100.00	1158	99
GH_07	*Isurus oxyrinchus*	Shortfin mako	0.0	99.83	1103	100
GH_08	*Isurus oxyrinchus*	Shortfin mako	0.0	95.09	898	100
GH_09	*Prionace glauca*	Blue shark	0.0	97.71	1051	100
GH_10	*Prionace glauca*	Blue shark	0.0	99.68	1153	100
GH_12	*Squatina aculeata*	Sawback angelshark	0.0	99.69	1168	99
GH_14	*Prionace glauca*	Blue shark	0.0	96.01	931	99
GH_16	*Carcharhinus signatus*	Night shark	0.0	98.83	1068	100
GH_17	*Sphyrna zygaena*	Smooth hammerhead	0.0	99.84	1140	100

GH_18	*Galeocerdo cuvier*	Tiger Shark	0.0	100.00	1014	100
GH_19	*Sphyrna zygaena*	Smooth hammerhead	0.0	100.00	1256	100
GH_20	*Prionace glauca*	Blue shark	0.0	99.69	1160	99
Other Ghanaian fish samples						
GH_30	*Scomber colias*	Atlantic chub mackerel	0.0	94.25	929	99
GH_31	*Pseudotolithus senegallus*	Law croaker	0.0	99.84	1149	99
GH_32	*Pseudotolithus senegallus*	Law croaker	0.0	99.39	1179	100
GH_33	*Pseudotolithus senegallus*	Law croaker	0.0	99.84	1153	99
GH_34	*Pseudotolithus senegallus*	Law croaker	0.0	91.93	848	99
GH_35	*Pseudotolithus senegallus*	Law croaker	0.0	100.00	1147	100
GH_36	*Pseudotolithus senegallus*	Law croaker	0.0	89.72	780	99
GH_37	*Pseudotolithus senegallus*	Law croaker	0.0	100.00	1164	99
GH_38	*Pseudotolithus senegallus*	Law croaker	0.0	99.84	1155	99
GH_39	*Pseudotolithus senegallus*	Law croaker	0.0	99.37	1147	98
GH_40	*Pseudotolithus senegallus*	Law croaker	0.0	99.84	1151	99
GH_42	*Sardinella maderensis*	Madeiran sardinella	0.0	100.00	1157	99
GH_43	*Sardinella maderensis*	Madeiran sardinella	0.0	100.00	1157	99
GH_44	*Sardinella maderensis*	Madeiran sardinella	0.0	99.68	1149	99
GH_45	*Sardinella maderensis*	Madeiran sardinella	0.0	99.68	1153	100

GH_46	*Sardinella maderensis*	Madeiran sardinella	0.0	99.52	1131	100
GH_47	*Sardinella maderensis*	Madeiran sardinella	0.0	100.00	1158	99
GH_48	*Sardinella lemuru*	Bali sardinella	0.0	99.05	1131	100
GH_49	*Engraulis encrasicolus*	European anchovy	0.0	99.84	1146	99
GH_50	*Engraulis encrasicolus*	European anchovy	0.0	100.00	1203	96
GH_51	*Engraulis encrasicolus*	European anchovy	0.0	99.85	1203	97
GH_52	*Engraulis encrasicolus*	European anchovy	0.0	100.00	1184	98
GH_53	*Engraulis encrasicolus*	European anchovy	0.0	99.85	1203	97
GH_54	*Engraulis encrasicolus*	European anchovy	0.0	100.00	1206	96
GH_55	*Engraulis encrasicolus*	European anchovy	0.0	100.00	1203	96
GH_56	*Engraulis encrasicolus*	European anchovy	0.0	99.85	1203	95
GH_57	*Engraulis encrasicolus*	European anchovy	0.0	100.00	1208	96
GH_58	*Engraulis encrasicolus*	European anchovy	0.0	100.00	1208	94
Asturian Shark samples						
SP_01	*Scyliorhinus canicula*	Small-spotted catshark	0.0	100.00	1092	100
SP_02	*Scyliorhinus canicula*	Small-spotted catshark	0.0	100.00	1092	100
SP_03	*Scyliorhinus canicula*	Small-spotted catshark	0.0	100.00	1110	100
SP_04	*Prionace glauca*	Blue shark	1E-141	100.00	514	100
SP_05	*Prionace glauca*	Blue shark	4E-110	100.00	409	100

SP_06	*Prionace glauca*	Blue shark	2E-118	100.00	436	100
SP_07	*Prionace glauca*	Blue shark	0.0	100.00	1109	99
SP_08	*Prionace glauca*	Blue shark	0.0	100.00	1050	99
SP_09	*Prionace glauca*	Blue shark	0.0	100.00	1110	100
SP_10	*Prionace glauca*	Blue shark	0.0	100.00	1110	100
SP_11	*Prionace glauca*	Blue shark	0.0	100.00	1110	100
SP_12	*Prionace glauca*	Blue shark	0.0	100.00	1092	100
SP_13	*Prionace glauca*	Blue shark	0.0	100.00	1092	100
SP_14	*Prionace glauca*	Blue shark	0.0	100.00	1110	100
